# Multiple probabilistic traveling salesman problem in the coordination of drug transportation—In the context of sustainability goals and Industry 4.0

**DOI:** 10.1371/journal.pone.0249077

**Published:** 2021-03-25

**Authors:** Sebastian Twaróg, Krzysztof Szwarc, Martyna Wronka-Pośpiech, Małgorzata Dobrowolska, Anna Urbanek

**Affiliations:** 1 Department of Social Logistics, University of Economics in Katowice, Katowice, Poland; 2 Institute of Computer Science, University of Silesia in Katowice, Katowice, Poland; 3 Department of Entrepreneurship and Management Innovation, University of Economics in Katowice, Katowice, Poland; 4 Department of Pedagogy, Silesian University of Technology, Gliwice, Poland; 5 Department of Transport, University of Economics in Katowice, Katowice, Poland; University of Defence in Belgrade, SERBIA

## Abstract

Improving the effectiveness of route planning, especially in road transport deliveries is a challenge we need to face in the context of advancing climate change and the sustainable development goals. The main aim of the paper is to demonstrate the above average and utilitarian significance of the multiple probabilistic traveling salesman problem (MPTSP) in the coordination and modeling of sustainable product transportation, which is a novelty at the theoretical, conceptual, methodological and empirical level. We propose a new, hybrid algorithm of solving MPTSP instances (it connects harmony search, *k*-means and 2-opt), which can be successfully used in economic practice for coordination and modeling of Industry 4.0. The effectiveness of proposed approach is tested using a case study of drugs distribution services and datasets obtained from the transportation enterprise located in Poland. The study focuses on the issue of planning routes, with particular emphasis on the changing demand of customers. It should be stressed that this work may be of interest to researchers but also to management practitioners. The value added of this research lies in the innovative modeling the coordination of sustainable drug transportation as an instance of MPTSP and proposing an effective method to solve it. The main research results confirm that proposed method contributes to overall sustainability of studied supply chain.

## Introduction

In the 18th century, the first industrial revolution brought significant changes in industry thanks to steam engines. The second industrial revolution was based on electric power and mass production, while the attributes of the third included the integration of information technology and computers in manufacturing. As the need to gain and sustain competitive advantage has always acted as the engine of the development of industry and industrial processes, the fourth industrial revolution is just around the corner.

The fourth industrial revolution, commonly referred to as “Industry 4.0”, is not just a new era of manufacturing. Rather, it is expected to lead to the next level of production, wherein machines will redefine how they communicate with each other and their environment as well as how they perform their individual functions [1]. The concept of Industry 4.0 developed by Kagermann et al. [2] links the virtual and the real worlds with an emphasis on engineering applications such as robotics, digitization, and automation. The main ideas and tools underlying this new era include cyber-physical systems [3], virtual and augmented reality [4], cloud computing [5], industrial additive manufacturing, the Internet of Things and Industrial Internet of Things [6], big data analytics [7], intelligent robots, autonomous drones, machine learning and artificial intelligence (AI) [8]. AI (i.e., automated decision-making and operations that can occur without human intervention [9]) in particular seems to fit perfectly with the challenges that arise in the consolidation of the fourth industrial revolution: analysis and filtration of huge amounts of data, interpretation, and suggestions for the most highly recommended course of action.

These technologies have seeped into the manufacturing industry, allowing it to better solve challenges linked to the increasing need for product personalization and customization, short production life cycles, higher expected quality, reduced development time, and faster time to market. Furthermore, technological advances have driven radical transformations of the status quo and have significantly influenced a wide range of areas: industry, technical standards, security, education, law, science, research, the labor market, the social system [10], and logistics [11]. The above-mentioned demand for highly individualized products and services has forced the logistics sector to adapt to an ever-changing environment [11]. A growing number of authors refer to concepts such as “Logistics 4.0” or “Smart Logistics”: situations wherein logistics flows are not only flexible but also optimized, technology driven, and adjustable in response to market changes. The key logistics activities of transport, inventory management, materials handling, supply chain structure, and information flow are affected in Logistics 4.0 [12, 13]. The drive towards Logistics 4.0 as an element of Industry 4.0 has also been pointed out by Barreto et al. [11], who suggested that an efficient and strong Logistics 4.0 must rely on (1) Resource Planning, (2) Warehouse Management Systems, (3) Transportation Management Systems, (4) Intelligent Transportation Systems, and (5) Information Security. Furthermore, Logistics 4.0 is under pressure to pay more attention to sustainability issues in order to address broader sustainability challenges [14]. Moreover, the demand for sustainability has created new requirements for the operations of logistics activities.

Given the above considerations, the objective of this paper is to demonstrate the above average and utilitarian significance of the multiple probabilistic traveling salesman problem (MPTSP) in the coordination and modeling of sustainable product transportation through route planning. This paper offers a new point of view by introducing the new, hybrid algorithm of solving MPTSP instances. Moreover, the conducted research has the advantage of combining theoretical insights with empirical data. The proposed approach was tested using a big dataset originating from an enterprise delivering drugs located in Poland. The conducted study complements the previous theoretical output concerning the MPTSP with the results of an analysis of this issue in the context of sustainability. We are facing the growing problems of climate changes and natural resources depletion that largely result from growing transport activity and inefficiencies in transport planning and coordinating. That is why, the main motivation for this study was to apply a new approach of solving MPTSP instances in the process of improving the sustainability and efficiency of supply chains.

The structure of the paper is as follows. After an introduction, we present a discussion of the underlying theoretical assumptions of the MPTSP. Second, the harmony search (HS) algorithm for the MPTSP is addressed. The research design and results are presented in the subsequent sections, while the last part of the paper offers conclusions and suggests future research directions.

## Theoretical background

### Sustainable transportation

Dynamic changes in the macro- and microeconomic environments, as well as unexpected revolutionary changes in the functioning of markets and societies (for example, the pandemic period [15]), pose a number of qualitatively new challenges in the supply chain field. Today, a major challenge is the integration of sustainability principles into the supply chain, taking a multidimensional (economic, environmental, and social impact) and multiscale (institutional, geographical, and temporal) approach [16]. Another challenge is the need to improve logistic processes, taking into account the modification of logistics management objectives [17], while a third is the increasingly important integration of environmental issues into logistics activities [18]. These conditions imply that traditional logistics activities (with a particular emphasis on transport) have begun to shape the reliability of logistics services differently than before.

Currently, in many organizations, a large portion of the logistics costs in the supply chain are determined by transportation [19]. The transportation sector has proven to be particularly difficult territory for the advancement of sustainability, understood as limiting depletion of resources to the rate at which they can be replenished or alternatives can be identified [20]. Current forms of transportation are not sustainable [21]: The transportation sector consumes non-renewable resources, such as energy, human and ecological habitats, atmospheric carbon-loading capacity, and individuals’ available time [20]. In particular, the dynamic global development of road transport is one of the main sources of environmental and noise pollution and, as a result, of deteriorating quality of life. Taking this into account, there is a clear need to improve the efficiency and effectiveness of product delivery and overall sustainability. Various strategies have been proposed to achieve more sustainable transportation, defined as transportation that satisfies current transport and mobility needs without compromising the ability of future generations to meet their own [22]. Other authors have pointed to the emergence of new goals for transportation decision-making and for mainstreaming alternative practices by reconfiguring existing networks [23] through optimizing transportation in more sustainable ways [24, 25]. Sustainable transportation can be considered by examining the sustainability of the transport system itself, focusing on the positive and negative aspects and externalities of traffic and transport as they exist now or in the near future [26]. Sustainable transportation implies finding a proper balance between current and future environmental, social, and economic concerns [27]. As pointed out by Steg and Gifford [26], a distinction can be made here between behavioral and technological strategies. Behavioral strategies focus on reducing the level of road transport use through psychological interventions—for example, by shifting to less polluting modes of transport, changing destination choices, altering habits and attitudes, combining trips, or traveling less [26, 28]. Technological strategies are instead focused on reducing the negative impact per vehicle and per kilometer [26], mainly through [29]:

- increasing the efficiency of the transport system via digitization, automation, deployment of new data analysis solutions, and optimization methods (also key elements of Industry 4.0); and
- the technological transition toward low- and zero-emissions vehicles, mostly in the field of vehicle design, and the use of alternative forms of energy for transportation.

Digitization in transportation and logistics is crucial in terms of sustainability, as it allows for more resource-efficient traffic management and optimization of existing transport networks.

Depending on the restrictions imposed by business practices, the traveling salesman problem (TSP) or vehicle routing problem (VRP; derived from the TSP) are often used to plan routes as part of a technological strategy. The VRP assumes that reducing the number of vehicles used and total distance traveled will reduce greenhouse gas (GHG) emissions [30], emissions of toxic and harmful substances, and noise pollution [26] and, as a result, will affect sustainable transportation. Due to various restrictions, it is necessary to develop more detailed variants of these optimization problems, which, when used in business practice, may affect the provision of sustainable transport according to the VRP. Looking for countermeasures for this type of phenomenon (increasingly common in operational practice), the authors draw attention to the potential of the MPTSP, which can be used to coordinate and model sustainable product distribution in various contexts, where the capacity of vehicles is not a real problem and the collection of recipients is constant, but it is not always necessary to visit each of them (modeling the drug transportation process). Significantly, in recent years, there has been a growing interest in questions about the critical role of methods for network and organizational coordination [31] in organizational performance [32]. Coordination is considered the act of managing interdependencies between activities [31] performed to achieve a goal [33].

Nowadays, although computers have enormous computing power, it is still not possible to accurately solve larger instances of some optimization problems that occur in business practice (e.g., job-shop scheduling, VRP, TSP). To overcome these difficulties, scientists have developed a number of approximate approaches, including metaheuristics that often draw inspiration from the real world. Such approaches usually arrive at good solutions within a short period of time, which enables the solving of even complex problems (e.g., the flexible containership loading problem for seaport container terminals [34] or the problem of planning milk-run tours for just-in-time production subject to hard time windows in congested road networks [35]).

Among the various approximate approaches used to solve TSP instances, we can distinguish between the use of population-based (e.g., ant colony system [36], the novel memetic genetic algorithm [37], and HS [38]) and single-solution–based metaheuristics (e.g., simulated annealing (SA) [39] and tabu search (TS) [39]). They are usually more effective than classic heuristics such as 2-opt or 3-opt algorithms or nearest-neighbor algorithms (NNAs), but at the cost of greater time complexity. In addition, metaheuristics often require the determination of control parameter values that affect the exploration and exploitation capabilities of the algorithm. Their proliferation can be explained by the “no free lunch” theorem [40], according to which there is no one metaheuristic that is best for all optimization problems. Therefore, it is necessary to determine the effectiveness of a technique for a specific issue.

Metaheuristics such as SA [24, 41], TS [42], and HS [43] are also used to solve VRP variants. Popular approaches to the design of algorithms adapted for the VRP include the hybridization of metaheuristics with other methods (e.g., Barma et al. [44] combined a discrete antlion optimization algorithm with 2-opt, while Zhou et al. [45] proposed a hybrid bat algorithm with path relinking). The use of mathematical programming methods (linear and dynamic programming) mentioned in [46] should also be considered. However, due to their significant computational complexity, they are not used for larger VRP instances.

An example of a metaheuristic that has achieved good results in probabilistic TSP (PTSP) instances is HS [47], which draws inspiration from the process of seeking harmony in music and has been used in the process of solving many optimization problems [48]. This, indicates that it may have potential for solving MPTSP instances, especially in Industry 4.0. In [47], HS was combined with 2-opt to improve the algorithm’s exploitation capability. Hybridization is often used when solving VRP instances (e.g., [44, 45]), demonstrating its possibly beneficial effects on the results for the MPTSP.

### Multiple probabilistic traveling salesman problem

The PTSP is an NP-hard problem [49] proposed in Jaillet’s doctoral dissertation [50] as a development of the classic TSP. In the TSP, for the directed graph *G* = (*V*, *A*) (*V* = {1,…,*n*} and *A* = {(*i*, *j*):*i*, *j* ∈ *V*}), with edge weights designated as *c*_*ij*_ (representing the distance between nodes *i* and *j*), it is necessary to designate a tour (consisting of all *n* nodes) of the minimum length [51]. Following [51], we denote the basic model of the TSP with the Miller, Tucker, and Zemlin formulation as follows (with binary *x*_*ij*_ variables equal to 1 if arc (*i*, *j*) belongs to the optimal solution and *u*_*i*_ variables are used to define the order in which vertex *i* is visited by the salesman):

$$\begin{array}{c} min\sum_{i=1}^{n}\sum_{j=1}^{n}c_{ij}x_{ij}, \end{array}$$ (1)

$$\begin{array}{c} x_{ij}\in \left\{0,1\right\},\;i,j=1,\ldots ,n, \end{array}$$ (2)

$$\begin{array}{c} u_{i}\in Z,\;i=2,\ldots ,n, \end{array}$$ (3)

$$\begin{array}{c} \sum_{j=1}^{n}x_{ij}=1,\;i=1,\ldots ,n, \end{array}$$ (4)

$$\begin{array}{c} \sum_{i=1}^{n}x_{ij}=1,\;j=1,\ldots ,n, \end{array}$$ (5)

$$\begin{array}{c} u_{i}-u_{j}+\left(n-1\right)x_{ij}\leq n-2,\;i,j=2,\ldots ,n, \end{array}$$ (6)

$$\begin{array}{c} 1\leq u_{i}\leq n-1,\;i=2,\ldots ,n. \end{array}$$ (7)

The PTSP additionally assumes that each *i* node is assigned a certain probability *p*_*i*_ of being visited by the traveling salesman. The aim of the optimization, therefore, is to designate such a tour a priori λ (consisting of all *n* nodes) to make its average length as short as possible. According to [52], the value of the objective function in PTSP may be written as:

$$\begin{array}{c} E\left[L_{\lambda }\right]=\sum_{S\subseteq V}p\left(S\right)L_{\lambda }\left(S\right), \end{array}$$ (8)

where *S* is a subset of *V*;*L*_λ_(*S*) is the length of the route, assuming that clients visited belong to *S* (in the order they appear in the itinerary); and *p*(*S*) is the probability that all clients in *S* are to be visited, amounting to:

$$\begin{array}{c} p\left(S\right)=\prod_{i\in S}p_{i}\prod_{i\in V\backslash S}\left(1-p_{i}\right). \end{array}$$ (9)

The expected length of the a priori route λ = (1, 2, …, *n*) amounts to:

$$\begin{array}{c} \begin{array}{cc} E[L_{\lambda }]= & \sum_{i=1}^{n}\sum_{j=i+1}^{n}c_{ij}p_{i}p_{j}\prod_{k=i+1}^{j-1}\left(1-p_{k}\right)+\\ & \sum_{i=1}^{n}\sum_{j=1}^{i-1}c_{ij}p_{i}p_{j}\prod_{k=i+1}^{n}\left(1-p_{k}\right)\prod_{l=1}^{j-1}\left(1-p_{l}\right), \end{array} \end{array}$$ (10)

where *c*_*ij*_ represents the weight of the edge connecting nodes at positions *i* and *j* on the route.

When considering the PTSP, two particular variants need to be addressed:

1. Where each of the nodes *i* is described with the probability of visiting *p*_*i*_ = 1, we have an instance of the classic TSP [49].
2. If *p*_*i*_ for each node *i* is the same (and amounts to *p*), then we have a homogeneous PTSP, which was used to model a problem of drug distribution in [47] (from the utilitarian point of view, pharmacies are characterized practically with the same probability of supply necessity).

This paper is an extension of research presented in [47]. Therefore, we will further analyze only the homogeneous PTSP, in which the value from the formula (10) can be written as follows [52]:

$$\begin{array}{c} E\left[L_{\lambda }\right]=p^{2}\sum_{r=0}^{n-2}{\left(1-p\right)}^{r}L_{\lambda }^{\left(r\right)}, \end{array}$$ (11)

where $L_{\lambda }^{\left(r\right)}\equiv \sum_{j=1}^{n}c_{\left(j,1+(j+r)\;mod\;n\right)}$ [47].

The MPTSP is an innovative combination of the PTSP and multiple TSP. The second problem is the generalization of the classic TSP, in which it is necessary to determine the routes for *z* salesmen who start and end their journey at the base [53]. It has found practical application in, for example, the minimization of off-grade production at multi-site multi-product plants [54]. Various metaheuristics have been used to solve it, such as the improved genetic algorithm and particle swarm optimization [55].

In case it is necessary to plan routes for companies distributing drugs to a greater number of pharmacies, there is a need to separate delivery points into fewer drivers in order to execute the task efficiently. For this reason, the PTSP was broadened to include the possibility of *k* existing delivery zones (which might be interpreted as areas subjected to a given driver or a company branch), hence obtaining the MPTSP. For each delivery zone *m* (*m* = 1, …, *k*) a disjoint set of *V*_*m*_ nodes is assigned, which are to be handled within one route λ_*m*_(*V*_*m*_ ⊆ *V* and *V*_1_∪*V*_2_∪…∪*V*_*k*_ = *V*). The task amounts to assigning of the minimum sum of the average lengths of routes λ_*m*_ consisting of particular nodes belonging to the given set *V*_*m*_.

## Our approach to solving MPTSP instances

### HS for PTSP

HS is a popular metaheuristic first proposed in Geem’s doctoral dissertation [56]. It assumes the existence of the structure *HM* (harmony memory), which includes *HMS* harmonies (solutions of the problem) composed of various pitches (components of the solution). Based on two parameters, *PAR* (pitch adjustment rate) and *HMCR* (HM consideration rate), there is a iterative generation of new harmonies, whose subsequent pitches are selected according to the following rules:

1. For a probability equal to *HMCR* ⋅ (1 − *PAR*) for the pitch in position *i*, the value is chosen from the pitches in position *i* in *HM*.
2. For a probability equal to *HMCR* ⋅ *PAR*, the modified pitch value is chosen in position *i* (using the parameter *bw* (Bandwidth), described in more detail in [57]).
3. For a probability equal to 1 − *HMCR*, a random acceptable value is chosen.

The created harmony is compared with the worst harmony located in *HM*. If it is characterized with the most favorable value of the objective function, it takes its place, after which *HM* elements are sorted (assuming that the best harmony is in the first spot and the worst in the last). The entire procedure is repeated through *IT* iterations.

In [47], an attempt was made to adjust HS for the efficient solving of PTSP instances (based on an approach intended for the asymmetric TSP proposed in [38]). The authors assumed that the pitches in the harmony correspond to the number of nodes to be visited by the traveling salesman and that the order of their occurrence indicates the sequence of travel. They also pointed out the lack of efficiency in the choice of pitch value based exclusively on its absolute position in the harmony (due to the nature of the problem) and replaced this operation by choosing the value of a new pitch from among the available nodes (i.e., those still not added to the new route), which occurred in harmonies already located in *HM* after the most recently added node to the currently created route. The choice is made based on the roulette wheel method in such a way as to facilitate choosing more closely located nodes; if all the analyzed nodes have already been added to the route, a pseudorandom choice is made from among the available nodes. Additionally, the possibility of modifying the pitch with *HMCR* ⋅ *PAR* probability was replaced with making a greedy move intended to choose the node located nearest to the most recently visited node (thus eliminating the need to use *bw*). A new parameter *R* was also introduced to avoid premature convergence by means of resetting *HM* elements (leaving only the best harmony) after a defined number of iterations, without replacing the worst solution located in *HM* (an analysis of this approach was presented in [58]). The efficiency of HS for PTSP was additionally increased by applying 2-opt in the solution provided by the algorithm (while originally the solution was referred to as hybrid HS, in this work it is identified as HS). The pseudocode of the algorithm is shown in Algorithm 1.

**Algorithm 1 The HS for PTSP pseudocode [47]**.

1: **function** HS(*HMS*, *HMCR*, *PAR*, *IT*, *R*, first city)

2:  *iterations* = 0

3:  *iterationsFromTheLastReplacement* = 0

4:  **for** *i* = 0;*i* < *HMS*;*i* + + **do**

5:   *HM*[*i*] = stochastically generate feasible solution

6:  **end for**

7:  Sort *HM*

8:  **while** *iterations* < *IT* **do**

9:   *H*[0] = first city

10:   **for** *i* = 1;*i* < *n*;*i* + + **do**       ⊳ *n*—number of cities

11:    Choose random *r* ∈ (0, 1)

12:    **if** *r* < *HMCR* **then**

13:     *list* = generate list containing vertices occurring after *H*[*i* − 1] in *HM*

14:     **if** *list*.*length* > 0 **then**

15:      *H*[*i*] = choose element ∈*list* according to the roulette wheel

16:     **else**

17:      *H*[*i*] = choose randomly available city ∉ *H*

18:     **end if**

19:     Choose random *k* ∈ (0, 1)

20:     **if** *k* < *PAR* **then**

21:      *H*[*i*] = find nearest and available city from *H*[*i* − 1]

22:     **end if**

23:    **else**

24:     *H*[*i*] = choose randomly available city ∉ *H*

25:    **end if**

26:   **end for**

27:   **if** *f*(*H*) is better than *f*(*HM*[*HMS* − 1]) **then**

28:    *HM*[*HMS* − 1] = *H*

29:    Sort *HM*

30:    *iterationsFromTheLastReplacement* = 0

31:   **else**

32:    *iterationsFromTheLastReplacement* + +

33:   **end if**

34:   **if** *iterationsFromTheLastReplacement* = *R* **then**

35:    **for** *i* = 1;*i* < *HMS*;*i* + + **do**

36:     *HM*[*i*] = stochastically generate feasible solution

37:    **end for**

38:    Sort *HM*

39:    *iterationsFromTheLastReplacement* = 0

40:   **end if**

41:   *iterations* + +

42:  **end while**

43:  *HM*[0] = 2-opt(*HM*[0])

44:  return *HM*[0]

45: **end function**

### *k*-means clustering algorithm

*k*-means clustering is a popular non-hierarchical clustering algorithm [59] and an unsupervised learning algorithm [60]. It assumes the allocation of objects to *k* classes defined in advance, through the following steps:

1. Initial division of objects into *k* clusters;
2. Appointment of centroid for each cluster;
3. Assignment of each object to the cluster with the closest centroid; and
4. Re-execution of step 2, if the stop condition is not satisfied.

The pseudocode of the implemented algorithm is shown in Algorithm 2.

**Algorithm 2 The *k*-means for MPTSP pseudocode**.

1: **function** k-means(*k*, *coordinates*)

2:  *randomCoordinates* = shuffle(*coordinates*)

3  **for** *i* = 0;*i* < *k*;*i* + + **do**

4:   *centroids*[*k*] = *randomCoordinates*[*k*]

5:  **end for**

6:  *assignedVertices* = assignVertices(*centroids*, *coordinates*)

7:  **do**

8:   *copiedVertices* = *assignedVertices*

9:   *centroids* = generateNewCentroids(*assignedVertices*, *k*)

10:   *assignedVertices* = assignVertices(*centroids*, *coordinates*)

11:  **while** *copiedVertices* ≠ *assignedVertices*

12:  return *assignedVertices*

13: **end function**

14:

15: **function** assignVertices(*centroids*, *coordinates*)

16:  **for** *i* = 0;*i* < *coordinates*.*length*;*i* + + **do**

17:   *c* = findClosestCentroid(*centroids*, *coordinates*[*i*])

18:   *verticesInCentroid*[*c*].*add*(*coordinates*[*i*]))

19:  **end for**

20:  return *verticesInCentroid*

21: **end function**

22:

23: **function** generateNewCentroids (*assignedVertices*, *k*)

24:  **for** *i* = 0;*i* < *k*;*i* + + **do**

25:   *sumX* = 0

26:   *sumY* = 0

27:   *noOfVertices* = *assignedVertices*[*i*].*length*

28:   **for** *j* = 0;*j* < *noOfVertices*;*j* + + **do**

29:    *sumX*+ = *assignedVertices*[*i*][*j*].*X*

30:    *sumY*+ = *assignedVertices*[*i*][*j*].*Y*

31:   **end for**

32:   *averageX* = *sumX*/*noOfVertices*

33:   *averageY* = *sumY*/*noOfVertices*

34:   *centroids*[*i*] = *coordinate*(*averageX*, *averageY*)

35:  **end for**

36:  return *centroids*

37: **end function**

### Hybrid of HS and *k*-means clustering algorithm for MPTSP

In our research, we took a two-stage approach to solving MPTSP instances:

1. In the first step, the pharmacies are divided into *k* areas, where *k* is determined on the basis of the abilities of the given enterprise. Handled points are allotted to the given cluster using *k*-means clustering, where the initial assignment is determined on the basis of pseudo-random choice of *k* pharmacies, creation of centroids with their coordinates, and subsequent assignment of the nearest nodes to them (to determine the distance between nodes, we used the haversine formula). The coordinates of the centroids in the subsequent iterations are determined based on the arithmetic mean of the coordinates of the nodes that make up the cluster. The algorithm stops at the moment that the next iteration does not change the assignment of nodes to any cluster.
2. Each cluster created in step 1 is treated as a separate PTSP instance being solved by a new HS instance, according to the approach proposed in [47] (described in Subsection *HS for PTSP*). This approach allows for the use of parallel computing, facilitating shorter computation time. The routes obtained in particular PTSP instances are a solution of an MPTSP instance (the sum of their objective function is a value of the objective function of the MPTSP).

**Fig 1:**
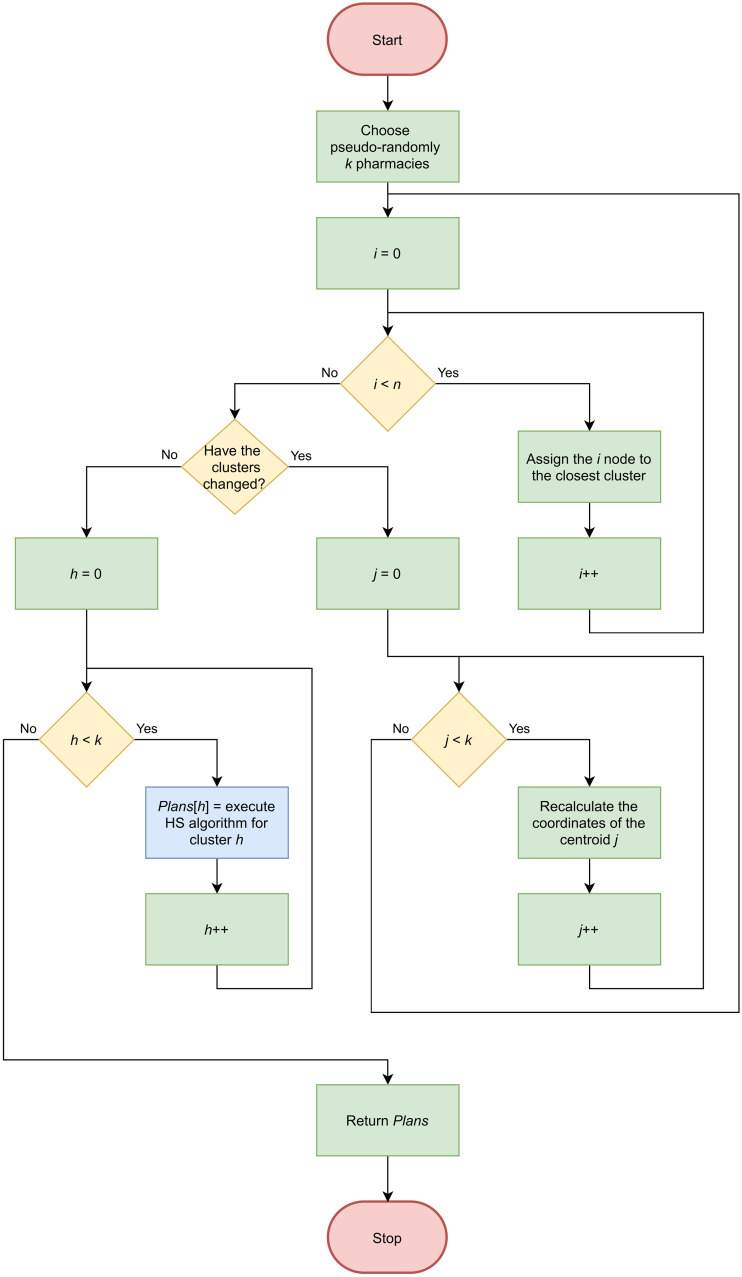
Flowchart of the proposed approach.

Fig 1 presents a flowchart of the proposed approach.

## Research methodology

Given the explanatory nature of the research, the usefulness and effectiveness of the proposed approach was checked using an instrumental case study [61], focusing on an enterprise delivering drugs within Silesian District, Poland. The problem instance consists of 90 nodes (i.e., 90 venues where drugs may be delivered), and the length between them was assigned using the haversine formula. In order to determine the usefulness of the method in economic practice, the study was performed with different parameter values: *k* (*k* ∈ {1, 2, 3, 4}) and *p* (*p* ∈ {0.75, 0.8, 0.9, 0.95, 1}). In addition, for the case where our problem can be reduced to a TSP instance (*p* = 1 and *k* = 1), we obtained an optimal result using the NEOS Server for Concorde [62]; the optimal length is 70.46 km (the result was obtained on the basis of a matrix containing distances rounded to six decimal places). According to [52], it is possible to calculate the theoretical lower bound to the homogeneous PTSP optimum using the following formula:

$$\begin{array}{c} LB=pL_{TSP}\left(1-{\left(1-p\right)}^{n-1}\right), \end{array}$$ (12)

where *L*_*TSP*_ is the optimal length of the corresponding TSP. Based on this, we determined the following *LB* values (for *k* = 1): 52.85, 56.37, 63.41 and 66.94 (for *p* = 0.75, *p* = 0.8, *p* = 0.9 and *p* = 0.95, respectively).

Due to the nondeterministic nature of the algorithm, the computations were repeated 30 times, using a different seed each time (but maintaining the same allocation of points to clusters for each *k*, in order to determine the impact of HS nondeterminism on results). The obtained value of the objective function was designated as *f* (with its average value designated as $\bar{f}$, sample standard deviation as *s*_*f*_, minimum value as *min*_*f*_, and maximum value as *max*_*f*_) and the actual time of execution of both steps of the method as *t* [s] (with its average value designated as $\bar{t}$, sample standard deviation as *s*_*t*_, minimum value as *min*_*t*_, and maximum value as *max*_*t*_).

Based on [47], we chose the following HS parameter values: *HMS* = 5, *HMCR* = 0.98, *PAR* = 0.25, *R* = 1000 and *IT* = 1, 000, 000.

We implemented the algorithms in *C*# using a Lenovo Y520 laptop with the following parameters: 32 GB RAM (SO DIMM DDR4, 2400MHz), Intel Core i7–7700HQ processor (4 cores, from 2.8-3.8 GHz; 6 MB cache) and Windows 10 Home 64-bit.

To verify the effectiveness of our approach, we compared the value of the objective function of the solutions constructed by HS with the results obtained using the 2-opt algorithm and NNA. Due to the significant influence of the initial solution on the results generated by 2-opt, the calculations were repeated 30 times, each time using a different pseudo-randomly generated initial solution. In a deterministic NNA, the generation of the solution started from the first node assigned to a given cluster. Due to the relatively low computational complexity of both methods, the execution time was not measured.

## Results

**Fig 2:**
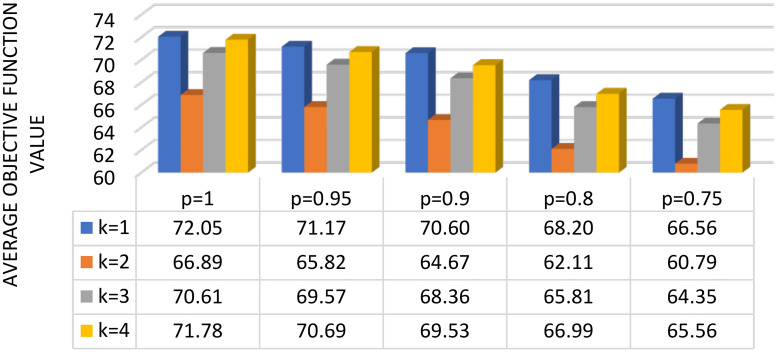
Summary of obtained average value of the objective function for different values of *k* and *p*.

**Fig 3:**
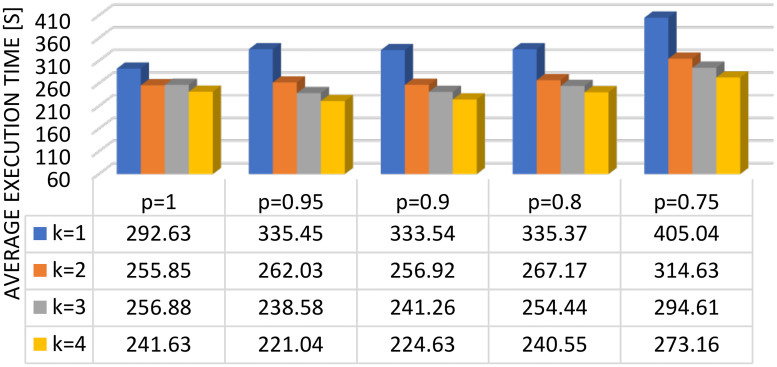
Summary of obtained average time of execution for different values of *k* and *p*.

The obtained results are presented in Tables 1–4 (for *k* = 1, *k* = 2, *k* = 3 and *k* = 4, respectively) and in Figs 2 and 3 (for $\bar{f}$ and $\bar{t}$, respectively). We found that—regardless of the value of the parameter *p*—the most beneficial variant for the analyzed case study was to make a division into two zones and prepare a separate route for each of them. The results were worse for *k* = 4 than for *k* = 2 and *k* = 3. For *k* = 3, the routes were less profitable than for *k* = 2, probably due to unfavorable assignment of points to zones by the *k*-means algorithm, which proves it is necessary to assign the appropriate value *k* to the given instance of the problem. A particularly noteworthy observation is that the greatest variability of the value *f* (*s*_*f*_) was characteristic of the results for *k* = 1, in which the non-deterministic nature of the method was emphasized, due to a relatively large number of nodes that make up the route constructed by HS (for instances where *k* = 4, assuming the creation of four relatively short routes, the impact of variability caused by the non-determinism of HS was insignificant, at the cost of a significant influence of the *k*-means algorithm).

**Table 1:** Summary of results for *k* = 1.

*p*	*min*_*f*_	*max*_*f*_	$\bar{f}$	*s*_*f*_	*min*_*t*_	*max*_*t*_	$\bar{t}$	*s*_*t*_
1	71.15	75.57	72.05	0.87	280.93	312.57	292.63	6.90
0.95	70.19	75.22	71.17	1.35	286.50	408.67	335.45	45.13
0.9	69.18	74.97	70.60	1.58	324.44	380.83	333.54	10.36
0.8	66.84	72.07	68.20	1.45	327.74	382.39	335.37	10.21
0.75	65.73	68.09	66.56	0.51	331.84	466.53	405.04	33.35

**Table 2:** Summary of results for *k* = 2.

*p*	*min*_*f*_	*max*_*f*_	$\bar{f}$	*s*_*f*_	*min*_*t*_	*max*_*t*_	$\bar{t}$	*s*_*t*_
1	66.46	67.59	66.89	0.24	222.26	312.59	255.85	31.75
0.95	65.40	66.36	65.82	0.24	249.30	317.91	262.03	18.83
0.9	64.23	65.33	64.67	0.24	250.55	309.79	256.92	10.77
0.8	61.87	62.43	62.11	0.14	249.38	360.49	267.17	27.27
0.75	60.54	61.20	60.79	0.16	286.70	351.25	314.63	18.12

**Table 3:** Summary of results for *k* = 3.

*p*	*min*_*f*_	*max*_*f*_	$\bar{f}$	*s*_*f*_	*min*_*t*_	*max*_*t*_	$\bar{t}$	*s*_*t*_
1	70.25	71.03	70.61	0.20	217.54	297.22	256.88	26.01
0.95	69.19	70.01	69.57	0.20	233.56	243.44	238.58	2.32
0.9	68.07	68.61	68.36	0.13	234.21	281.12	241.26	9.70
0.8	65.61	66.20	65.81	0.13	234.31	335.03	254.44	26.95
0.75	64.21	64.53	64.35	0.07	272.41	351.95	294.61	18.70

**Table 4:** Summary of results for *k* = 4.

*p*	*min*_*f*_	*max*_*f*_	$\bar{f}$	*s*_*f*_	*min*_*t*_	*max*_*t*_	$\bar{t}$	*s*_*t*_
1	71.74	72.06	71.78	0.06	213.09	274.98	241.63	23.00
0.95	70.64	70.70	70.69	0.02	215.49	225.60	221.04	2.48
0.9	69.48	69.69	69.53	0.05	218.26	259.36	224.63	8.58
0.8	66.94	67.03	66.99	0.04	219.45	307.96	240.55	25.54
0.75	65.54	65.66	65.56	0.05	250.15	327.15	273.16	17.84

When analyzing the actual time needed to execute the method, particular attention should be paid to the influence of external factors on the executed measurements. The decreased running time of the algorithm related to increases in the value of the parameter *k* was likely caused by the acceleration of the most time-consuming part of the two-stage procedure (i.e., the execution of HS). The limitation of the number of pitches included in particular harmonies (decreasing it to the cluster size) accelerated the execution of the main loop of HS. Consequently, the total time of sequential execution of HS instances for particular subsets of nodes was shorter than the time of execution of the method for *k* = 1. The increase of the execution time of the method for *p* = 0.75 with reference to the remaining values of *p* was likely caused exclusively by external factors affecting the measurement. However, it is particularly noteworthy that the running time of the entire algorithm was insignificant (and can be additionally shortened using parallel computations for *k* > 1), enabling designation of the solution within 4–7 minutes on average. This makes the use of this technique possible in many cases occurring in contemporary economic practice.

**Fig 4:**
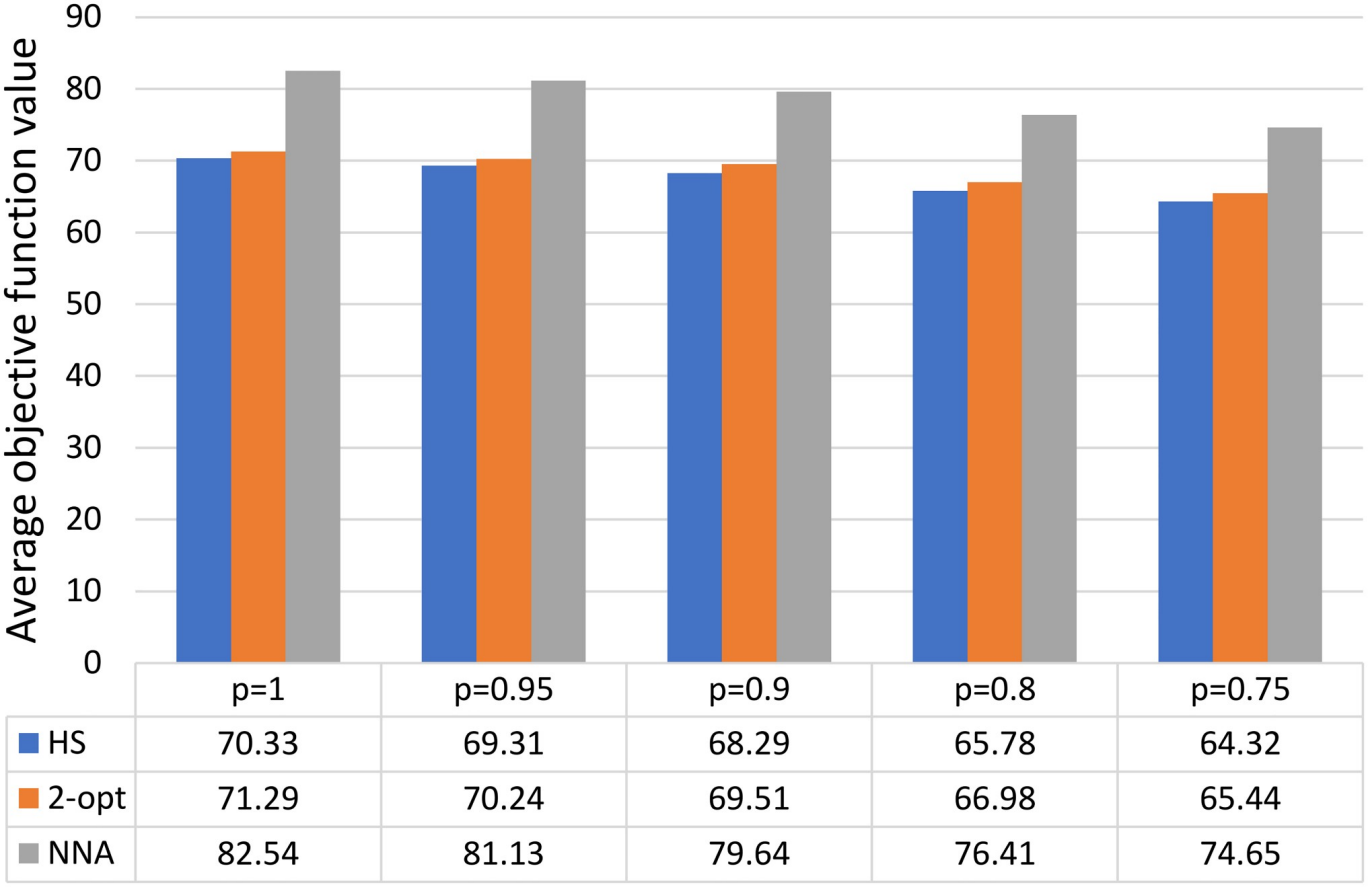
Comparison of the obtained value of the objective function with the results obtained using other methods.

Fig 4 presents a comparison of the average value of the objective function obtained via HS with the results determined by NNA and 2-opt (average result calculated on the basis of the average objective function value for *k* ∈ {1, 2, 3, 4}). We found that our approach was more effective than the classic heuristics adapted to the TSP and enables the construction of shorter routes.

## Discussion, conclusions, and future work

In this work, we proposed a hybrid algorithm for solving MPTSP instances (combining HS, *k*-means clustering, and 2-opt). It can be successfully used in economic practice for coordination and modeling in Industry 4.0 and in applications with special restrictions that prevent the use of popular VRP and TSP variants (e.g., to optimize itineraries in the process of distribution of drugs to pharmacies). Our algorithm allowed us to obtain acceptable results in a relatively short amount of time. In addition, its construction allows for a simple application of parallel computations, facilitating an additional increase in efficiency. We compared the obtained average results for the variant with *k* = 1 (72.05, 71.17, 70.60, 68.20 and 66.56 for *p* = 1, *p* = 0.95, *p* = 0.9, *p* = 0.8 and *p* = 0.75, respectively) with the optimal result determined in Section *Research Methodology* (70.46 for *p* = 1) and specific *LB* values (66.94, 63.41, 56.37 and 52.85 for *p* = 0.95, *p* = 0.9, *p* = 0.8 and *p* = 0.75, respectively). We showed that our proposed method can contribute to the overall sustainability of the supply chain. In light of our close-to-optimal results, we can state that the application of the algorithm in business practice will reduce the average length of travel routes. When comparing the results with the solutions obtained by NNA and 2-opt, we found that our approach is more effective than the classic heuristics adapted to the TSP and enables the construction of shorter travel routes. The reduction of distance travelled by vehicles means a reduction in GHG emissions; emissions of toxic and harmful substances polluting air, water, and soil; noise pollution; and damage to natural habitats. It is of especially great importance for drug deliveries, which are mostly based on road transport—the mode of transportation with the greatest negative impact on the environment. Moreover, a decrease in the distance traveled by vehicles means more efficient traffic flows; as such, a reduction in the number of vehicles used results in decreased congestion and resource consumption across the overall transportation system.

Shifting towards Industry 4.0 is a response to the need to establish a sustainable economy, especially in the context of the growing problems of climate change and natural resources depletion. In this context, optimization and digitization are key elements of energy- and resource-efficient logistics. Studies conducted to date show that in many sectors—particularly the transport sector—technology itself is not an issue anymore, in light of the ability to access enormous computing power and data sets. Despite this, however, it is still not possible to solve some optimization problems in business practice. Accordingly, new approaches like the MPTSP are needed.

Conscious implementation of behavioral and technological strategies may improve environmental quality, urban quality of life, and destination accessibility [26]. In terms of technological strategies, the rapid growth of Logistics 4.0 as an element of Industry 4.0 will lead to a wider variety of available tools. However, it should be kept in mind that sustainable transportation (planning) may require a change in people’s mindsets at the same time.

Based on our results, the choice of the number of clusters that must be determined using the proposed method should be designated not only on the basis of the resources available to the enterprise in question but also in light of the nature of the given task. In addition, when considering only the lengths of routes existing in particular clusters, the distance from the base of the enterprise delivering drugs is not analyzed. This must be taken into account in practical applications, as it requires that travel commence at a given location (a factor that could affect the optimal number of clusters). When solving the instance of the problem, the actual distances between nodes should be applied, taking into account the nature of the existing line infrastructure.

As indicated, an important new theoretical implication of our work is the study of the MPTSP in the coordination and modeling of sustainable product transportation. Moreover, practitioners can use these research results to improve and optimize the flow of materials in a more sustainable way. The proposed approach can also be modified and adapted to optimize other kinds of supply chains as well as developed for the purposes of further research work. Further work on this study’s subject matter might include extensions of the analyzed “test bed” (in order to analyze the behavior of the algorithm on diversified data) and increasing the efficiency of the algorithm itself. The first step of the procedure should include modifying the *k*-means algorithm, according to other studies, such as [59, 63] in which the quality of the obtained results was improved by applying the appropriate procedure for selecting initial solutions. [64] proposed using a modification that enabled the time of *k*-means execution to be shortened. The hybrid HS algorithm used to execute the second step of the procedure in this work can be modified with a more efficient version of 2-opt (also referenced in [47], proposing the use of 2-p-opt, used in the paper [49]) that can be combined with other metaphor-based metaheuristics or swarm intelligence algorithms (e.g., it was combined with Artificial Bee Colony [65] and with Cuckoo Search in [66]) and may apply dynamically changing parameter values (such as *PAR*, used in [67]). Additionally, it would be worthwhile to analyze the impact of the values of particular parameters on the method’s efficiency. It may also prove useful to formulate a compact mathematical model with objective functions and constraints for the MPTSP in order to use appropriate solvers. Finally recoverable robustness concepts [68, 69] can be integrated into the PTSP.

The limitations of this study result from the necessity to limit the volume of the paper and discussed issues. The presented empirical analysis does not include the actual calculation of the environmental impact resulting from the application of the developed hybrid algorithm. The reduction of negative impact on the environment resulting from the reduction of time and fuel consumption, noise and air pollutant emission etc. can be dimensioned in appropriate values and monetised, which in fact would allow us to measure the effectiveness of the proposed approach and the real contribution to sustainability. In this regard, the conducted study may be the basis for designing future research.

## Supporting information

S1 File Distance matrix. The distance matrix used in the research.
(TXT)
